# Revealing Lingonberry’s Neuroprotective Potential in Alzheimer’s Disease Through Network Pharmacology and Molecular Docking

**DOI:** 10.3390/ijms26052363

**Published:** 2025-03-06

**Authors:** Juncheng Li, Mian Wang, Yi Wang, Xichen Peng, Guixiang Lv, Tianhu Zheng, Yahui Peng, Jihong Li

**Affiliations:** 1 Department of Biochemistry and Molecular Biology, School of Basic Medicine, Harbin Medical University, Harbin 150086, China; junchengli47@gmail.com (J.L.); 13045145687@163.com (M.W.); 13896776640@163.com (Y.W.); pengxichen947@gmail.com (X.P.); lvgx@hrbmu.edu.cn (G.L.); zhengtianhu188@163.com (T.Z.); 2 Translational Medicine Center of Northern China, Harbin 150000, China

**Keywords:** lingonberry, Alzheimer’s disease, ferulic acid, monoamine oxidase B, network pharmacology, molecular docking, molecular dynamics

## Abstract

Alzheimer’s disease is a progressive neurodegenerative disorder with limited treatment options. Lingonberry (*Vaccinium vitis-idaea* L.) has demonstrated neuroprotective and anti-inflammatory properties, yet the underlying mechanisms remain unclear. This study employed network pharmacology, molecular docking, and molecular dynamics simulations to explore the therapeutic potential in Alzheimer’s disease. Pathway analysis identified monoamine oxidase B as a key target involved in serotonergic synapse dysfunction related to Alzheimer’s disease. Molecular docking revealed that ferulic acid, a major bioactive compound in lingonberry, exhibits strong binding affinity to monoamine oxidase B. Further molecular dynamics simulations confirmed the stability of this interaction, highlighting the potential inhibitory effect of ferulic acid on monoamine oxidase B. These findings provide novel insights into the neuroprotective mechanisms of lingonberry and suggest its potential as a natural therapeutic intervention for Alzheimer’s disease.

## 1. Introduction

Alzheimer’s disease (AD) is a progressive neurodegenerative disorder characterized by cognitive decline, memory impairment, and loss of daily functioning [1]. At present, more than 50 million people in the world are diagnosed with AD [2]. In the AD brain, the accumulation of amyloid β (Aβ) peptides begins 15 to 20 years before the onset of clinical symptoms, making it one of the earliest pathological events in disease progression [3]. The primary pathological hallmarks of AD include Aβ deposition, tau protein hyperphosphorylation, and chronic neuroinflammation [4]. The overexpression of monoamine oxidase B (MAO-B) plays a significant role in AD progression [5]. Studies have shown that MAO-B is upregulated in key brain regions of AD patients, such as the frontal cortex, hippocampal CA1 region, and entorhinal cortex, where it promotes Aβ production and accumulation, thereby accelerating neuronal damage [6]. Consequently, targeting MAO-B may serve as an effective strategy to modulate Aβ pathology, offering new therapeutic avenues for AD treatment.

Emerging research indicates that metabolic disorders, including type 2 diabetes, obesity, and hypertension, are closely associated with AD pathogenesis [7,8,9]. Impaired glucose metabolism, mitochondrial dysfunction, and oxidative stress disrupt insulin signaling, promoting Aβ aggregation and neurodegeneration [10]. Moreover, blood–brain barrier (BBB) integrity is compromised by hypertension, accelerating Aβ accumulation, and tau pathology progression [11]. Therefore, controlling metabolic risk factors may be a promising approach for the prevention and intervention of AD [12].

Polyphenols (PPs), a diverse class of secondary plant metabolites, exhibit antioxidant, anti-inflammatory, and neuroprotective properties [13]. Structurally, PPs contain one or more hydroxyl groups attached to an aromatic ring, classifying them into phenolic acids, flavonoids, stilbenes, and lignans [14]. Recent studies have shown that PPs can modulate metabolic pathways, including glucose and lipid metabolism, by regulating gene expression [15]. PPs offer promising strategies for AD prevention and treatment by targeting multiple pathological mechanisms, including Aβ aggregation, tau pathology, oxidative stress, and neuroinflammation [16]. Additionally, PPs can improve insulin sensitivity, reduce blood pressure, and lower blood glucose levels, thereby mitigating metabolic risk factors associated with AD [17]. Ferulic acid, a widely studied phenolic acid, has been shown to reduce oxidative stress and inhibit neuroinflammation [18].

This study investigated the potential role of lingonberry (*Vaccinium vitis-idaea* L.), a polyphenol-rich berry [19], in AD prevention. Through network pharmacology, molecular docking, and molecular dynamics simulations, key bioactive compounds were identified and their interactions with MAO-B were determined, providing novel insights into the multi-target mechanisms of lingonberry in AD. Furthermore, ferulic acid was highlighted as a potential MAO-B modulator, offering a new perspective on its therapeutic application in AD management.

## 2. Results

### 2.1. Differential Gene Analysis

**Figure 1:**
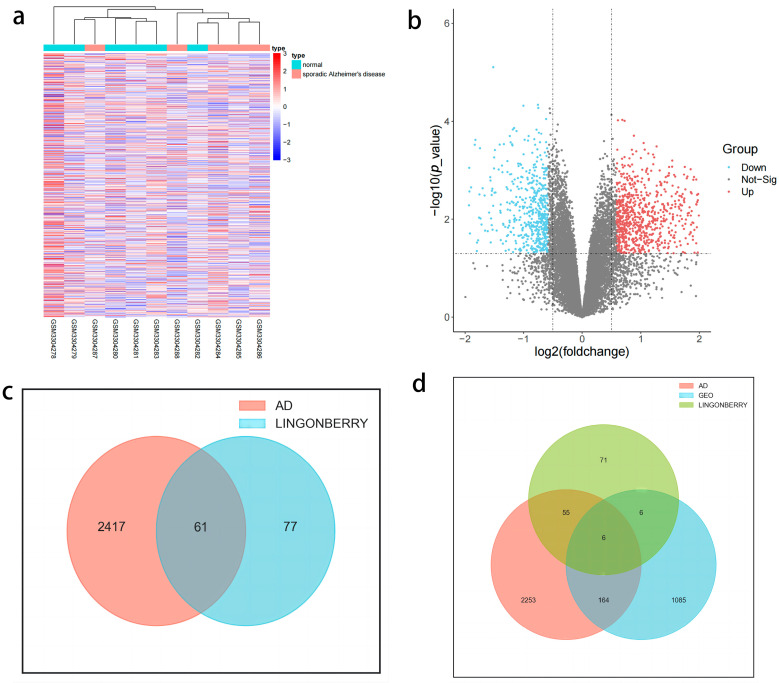
(**a**) Heat map of GSE117585 differential gene analysis results. (**b**) Volcanic map of GSE117585 differential gene analysis results. The interrupted vertical lines at log_2_(FC) = ±1 indicate a 2-fold change, and the interrupted horizontal line at −log_10_(*p*) = 1.3 corresponds to *p* = 0.05. Genes lying beyond both thresholds are considered significantly up- or down-regulated. (**c**) The intersection of potential targets of active components in lingonberry and AD target genes. (**d**) The intersection of potential targets of active components in lingonberry with AD target genes and differential genes of GSE117585.

Through interrogation of five leading databases—PharmGKB (https://www.pharmgkb.org/ accessed on 26 February 2024), OMIM (https://www.omim.org/ accessed on 26 February 2024), GeneCards (https://www.genecards.org/ accessed on 26 February 2024), DisGeNET, and CTD (https://ctdbase.org/ accessed on 26 February 2024)—we identified a cohort of 2478 target genes associated with AD: 63 from PharmGKB, 546 from OMIM, 1943 from GeneCards, 220 from DisGeNET, and 117 from CTD. We sourced gene expression profile data related to AD from the GEO database (https://www.ncbi.nlm.nih.gov/gds accessed on 26 February 2024) through the GSE117585 dataset. The platform for GSE117585 was the [HG-U133_Plus_2] Affymetrix Human Genome U133 Plus 2.0 Array [CDF: Brainarray HGU133Plus2_Hs_ENSG_22], which included five samples from patients with sporadic Alzheimer’s disease (SAD) and six control samples (NL). Analysis revealed 1261 differentially expressed genes in GSE117585, comprising 737 upregulated and 524 downregulated genes (Figure 1a,b). First, the disease targets from the AD database were intersected with drug targets for GO, KEGG, and PPI analysis, aiming to explore potential targets of the active components of lingonberry with a larger dataset. This intersection identified 61 genes (Figure 1c). Then, the GEO results were intersected again to facilitate molecular docking and identify the most suitable active compounds. This intersection identified 6 core intersecting genes (Figure 1d)—*MAO-B*, *PREP*, *CA2*, *CNR1*, *KDR*, and *NR3C2*—which were likely to play crucial roles in the interaction between lingonberry-derived active compounds and AD-related pathways, thereby helping us identify the most relevant targets for further validation.

### 2.2. PPI Network Construction

**Figure 2:**
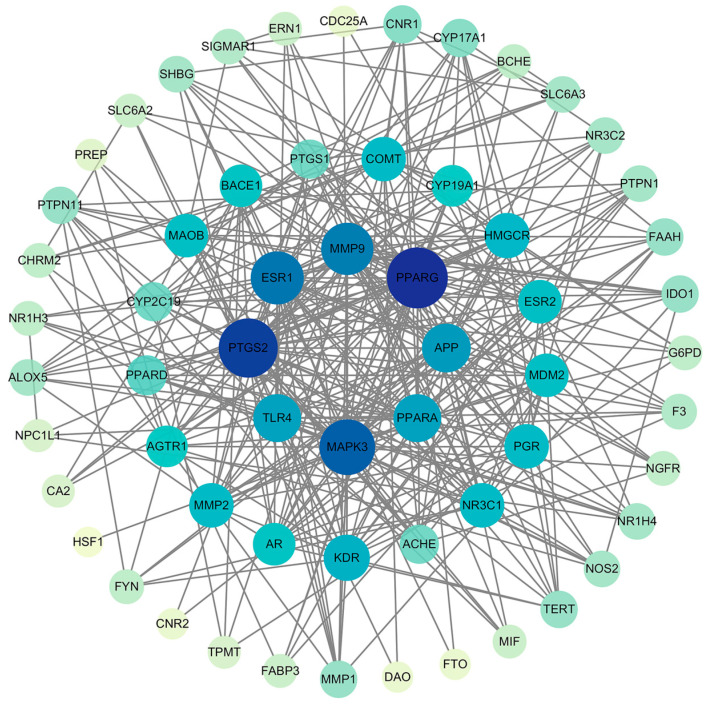
PPI analysis was conducted on the 61 overlapping targets using the STRING database and visualized in Cytoscape.

The 61 overlapping targets were integrated into the STRING platform, which facilitated the generation of an associated protein–protein interaction (PPI) network. The node color represented the degree of centrality, with darker nodes indicating highly connected hub proteins, such as PPARG, MAPK3, PTGS2, and MAOB. We then applied the MCODE plugin to analyze this PPI network (Figure 2). After the analysis, we directed our attention to the gene clusters that were forming minor subnetworks. Of particular interest was the formation of a compact yet intricate subnetwork comprising MAOB, ACHE, and FAAH within the broader network. We hypothesize that these compact structures may be indicative of distinct biological roles or involvement in specialized biological processes.

### 2.3. GO Functional Annotation and KEGG Pathway Enrichment Analysis Results

**Figure 3:**
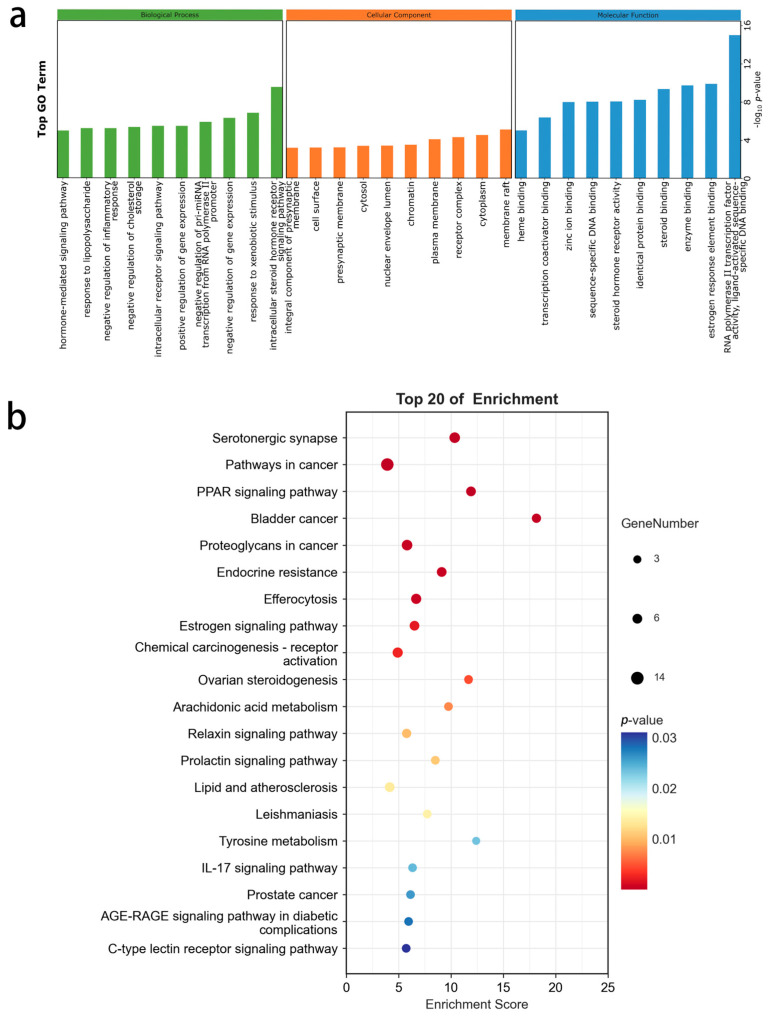
(**a**) The GO analysis of the intersected genes (molecular function, cellular component, biological process). (**b**) KEGG enrichment analysis of the intersected genes.

We used the DAVID database to perform GO functional and KEGG pathway enrichment analysis on 61 intersecting genes (Figure 3a,b). The GO functional annotation results of the targets indicate involvement in 217 BP functions, with lingonberry’s neuroprotective effects mainly acting on intracellular steroid hormone receptor signaling (GO:0030518). In the CC category, we mainly enriched the membrane raft (GO:0045121), cytoplasm (GO:0005737), and receptor complex (GO:0043235). In the MF category, RNA polymerase II transcription factor activity (GO:0000981) and estrogen response element binding (GO:0034056) were the main enriched items. We also conducted a detailed KEGG analysis, where the targets were primarily enriched in 37 related signaling pathways, with Serotonergic synapse (hsa04726) as the main enriched item. After conducting PPI analysis and processing with the MCODE plugin, we paid special attention to the genomes forming small subnetworks. In the KEGG analysis of the serotonergic synapse pathway, several biological processes related to 5-HT were involved, and MAO inhibitors can block these metabolic processes, reducing the degradation of 5-HT and increasing its concentration in the synaptic cleft. Additionally, several studies have reported that the overexpression of MAO-B in the brains of AD patients is associated with cognitive dysfunction [20].

Taken together with the findings from differential gene analysis and PPI network construction, we selected MAO-B as the primary target for further investigation. Given its involvement in serotonergic synapse dysfunction, we paid particular attention to the *MAO-B* gene.

### 2.4. The “Component–Target–Disease (CTD)” Network

**Figure 4:**
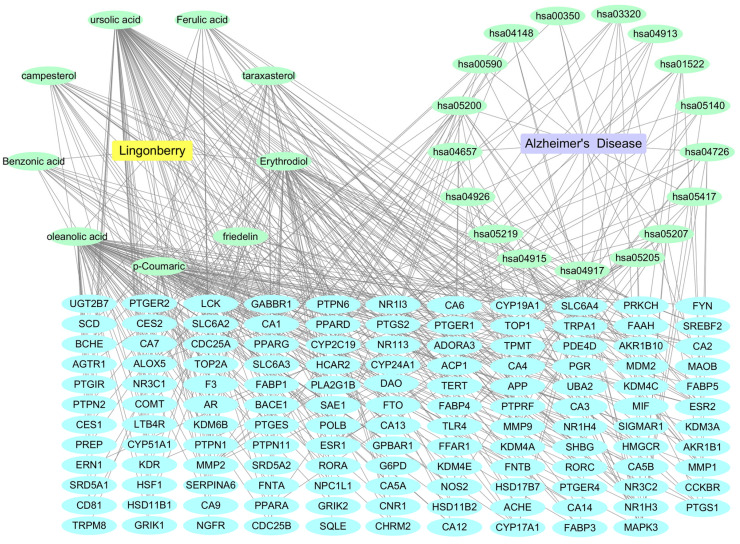
The component–target–disease network which is illustrating the interactions between nine key components of lingonberry and the top 17 AD-related KEGG pathways.

We used Cytoscape version 3.8.1 software to construct a network of lingonberry’s nine components and the top 17 AD-related KEGG pathways (Figure 4). The network emphasizes highly connected nodes and critical interaction edges, revealing the potential roles of these compounds in modulating AD-related biological processes. The updated figure legend further elucidates these key features, thereby clarifying the molecular mechanisms underlying their therapeutic effects in AD.

### 2.5. Molecular Docking Protocol

In this study, we aimed to identify small molecules from lingonberry that interact with MAO-B through molecular docking. Four candidate compounds—ferulic acid, benzoic acid, erythrodiol, and p-coumaric acid—were selected based on their presence in the intersection of the gene set, and their three-dimensional (3D) structures were obtained from PubChem, along with their two-dimensional (2D) structures for further visualization. Molecular docking was performed using AutoDockTools (version 1.5.7) to assess the binding energies and hydrogen bond interactions between these compounds and MAO-B. The docking results indicated that ferulic acid formed three hydrogen bonds, with a binding energy of −5.26 kcal/mol; benzoic acid did not form hydrogen bonds, with a binding energy of −4.96 kcal/mol; erythrodiol, despite not forming hydrogen bonds, exhibited the strongest binding affinity among the tested compounds, with a binding energy of −7.78 kcal/mol; and p-coumaric acid formed three hydrogen bonds, with a binding energy of −5.01 kcal/mol. To further analyze the ligand–protein interactions, PyMOL (version 2.6) was used for visualization, mapping the binding sites of the small molecules within the MAO-B active site. Additionally, the RMSD value for ferulic acid was calculated as 1.146 Å, indicating a stable binding conformation (Table 1).

**Figure 5:**
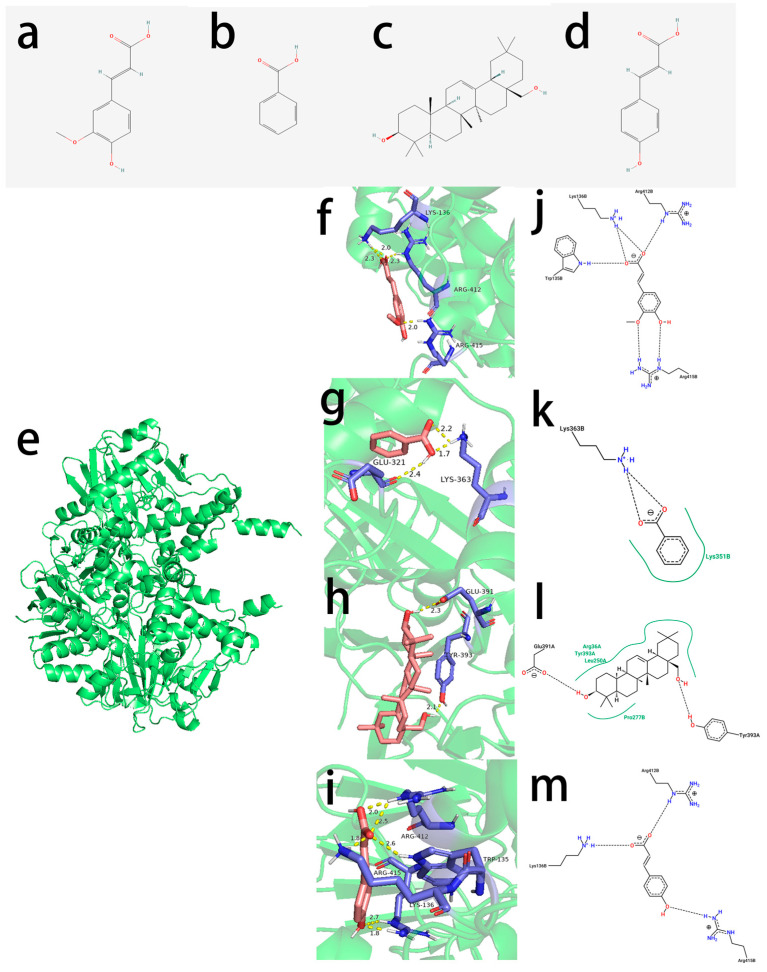
Two-dimensional chemical structures of (**a**) ferulic acid, (**b**) benzonic acid, (**c**) erythrodiol, and (**d**) p-coumaric acid, red represents oxygen atoms, and blue represents hydrogen atoms. (**e**) Interaction mechanisms and binding modes of protein MAO-B inhibitors. Three-dimensional close view into the binding mode of (**f**) ferulic acid, (**g**) benzonic acid, (**h**) erythrodiol, (**i**) p-coumaric acid, blue indicates the structural components of the residue, and red is used to highlight key interaction sites. Three-dimensional interaction plots showing the binding interactions of (**j**) ferulic acid, (**k**) benzonic acid, (**l**) erythrodiol, and (**m**) p-coumaric acid with the catalytic and binding residues of the studied receptors.

To validate the docking protocol, we selected selegiline, a known MAO-B inhibitor, as a positive control. The docking results showed that selegiline exhibited a binding energy of −8.12 kcal/mol, forming four hydrogen bonds with an RMSD of 0.98 Å, confirming its strong binding affinity to the active site of MAO-B. Concurrently, benzoic acid, which has a binding energy of −4.96 kcal/mol (higher than −5 kcal/mol), was selected as an internal negative control to assess the binding efficacy of the candidate molecules to MAO-B. To enhance the visualization of ligand–receptor interactions, PoseView was used to generate 2D interaction diagrams, illustrating the key catalytic residues involved in ligand binding (Figure 5). Given ferulic acid’s favorable binding energy, multiple hydrogen bond formations, and structural stability, we propose that ferulic acid exhibits strong binding potential with MAO-B, warranting further investigation into its inhibitory effects.

### 2.6. Molecular Dynamics Protocol

**Figure 6:**
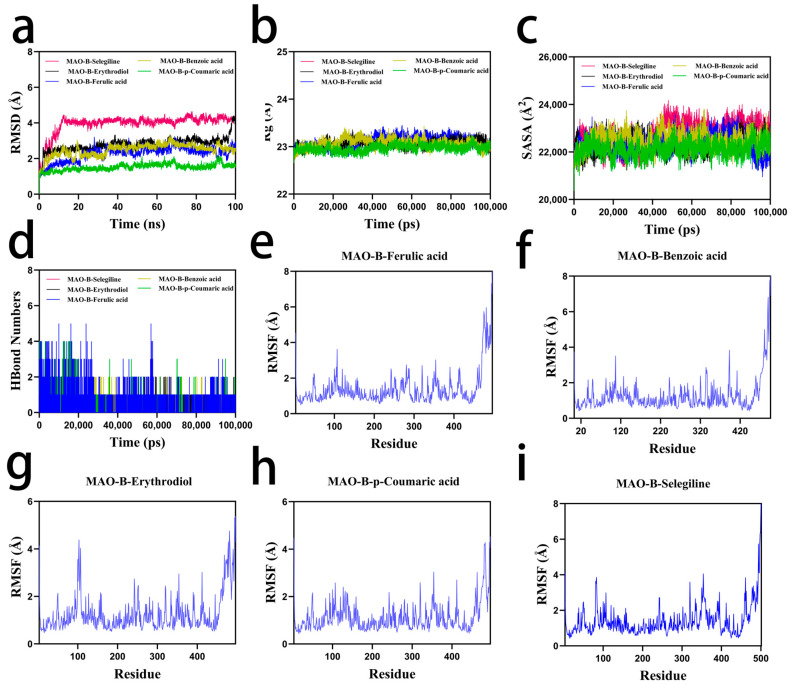
(**a**) RMSD analysis of protein–ligand complexes. (**b**) Radius of gyration (Rg) analysis of protein–ligand complexes. (**c**) Solvent-accessible surface area (SASA) analysis of protein–ligand complexes. (**d**) Hydrogen bond analysis of protein–ligand complexes. Root mean square fluctuation (RMSF) analysis of (**e**) MAO-B–ferulic acid, (**f**) MAO-B–benzoic acid, (**g**) MAO-B–p-coumaric acid, (**h**) MAO-B–erythrodiol, and (**i**) MAO-B–selegiline.

RMSD serves as a robust metric to evaluate conformational stability and positional deviations of protein–ligand complexes from their initial configurations. Lower RMSD values indicate enhanced conformational stability. All systems (MAO-B–ferulic acid, MAO-B–benzoic acid, and MAO-B–p-coumaric acid) reached equilibrium after 10 ns, with RMSD fluctuations stabilized at 2.5 Å, 2.7 Å, and 1.5 Å, respectively. The MAO-B–erythrodiol complex exhibited stable fluctuations between 5–95 ns, followed by a minor upward trend post 95 ns, yet remained within a 3 Å threshold. In contrast, the MAO-B–selegiline complex, used as a positive control, reached equilibrium at 15 ns, maintaining RMSD fluctuations around 4.2 Å (Figure 6a). Slight variations in the radius of gyration (Rg) and solvent-accessible surface area (SASA) values were observed for all complexes (MAO-B–ferulic acid, MAO-B–benzoic acid, MAO-B–p-coumaric acid, MAO-B–erythrodiol, and MAO-B–selegiline), suggesting moderate conformational adjustments during the simulation. (Figure 6b,c).

Hydrogen bonding critically influences ligand–protein interactions. The MAO-B–ferulic acid complex displayed 0–5 hydrogen bonds (predominantly 2), while MAO-B–benzoic acid exhibited 0–2 bonds (primarily 1). MAO-B–p-coumaric acid maintained 0–4 bonds (average 2), and MAO-B–erythrodiol showed 0–3 bonds (average 1). Meanwhile, the MAO-B–selegiline complex formed 0–1 hydrogen bond (average 1) (Figure 6d).

Root mean square fluctuation (RMSF) analysis revealed low flexibility (≤2 Å) across all complexes, indicating high structural stability. Notably, MAO-B–ferulic acid demonstrated the lowest residue-level fluctuations. All systems exhibited favorable binding stability during molecular dynamics (MD) simulations. MAO-B–ferulic acid outperformed others, maintaining consistent hydrogen bonding (average 2), low RMSF values (≤2 Å), and stable RMSD fluctuations (~2.5 Å post equilibrium). In contrast, MAO-B–benzoic acid and MAO-B–p-coumaric acid displayed fewer hydrogen bonds and marginally higher RMSD values. Although MAO-B–erythrodiol remained below 3 Å RMSD, its post-95 ns upward trend suggested slightly inferior stability relative to ferulic acid (Figure 6e–h).

Consistent with molecular docking (binding energy: −5.26 kcal/mol; 3 hydrogen bonds), MD simulations further confirmed the strong binding stability of ferulic acid, reinforcing its potential as a natural MAO-B inhibitor. The inclusion of selegiline as a positive control provided a reliable reference, ensuring the credibility of our simulation approach. In summary, these findings highlight the potential of natural bioactive compounds as MAO-B inhibitors, warranting further investigation into their therapeutic implications for neurodegenerative diseases.

## 3. Discussion

In the network pharmacology process of this study, we analyzed the GSE117585 dataset, which helped further clarify the AD-related genes and their expression profiles. In the KEGG analysis, eight genes were enriched in the serotonergic synapse signaling pathway, *APP*, *MAOB*, *ALOX5*, *CYP2C19*, *PTGS2*, *SLC6A4*, *MAPK3*, and *PTGS1*, demonstrating changes in AD-related to serotonergic synapses. The efferocytosis and IL-17 signaling pathways also demonstrated how lingonberry influences AD through inflammation and apoptosis. MAO-B plays a crucial role in AD progression by influencing Aβ production via γ-secretase modulation [5]. Currently, several drugs are being developed to treat AD by inhibiting MAO-B, but they are still in the early stages, such as Ladostigil, which is still undergoing Phase IIb clinical trials [21]. Rasagiline is used for the treatment of Parkinson’s disease, but its efficacy in treating AD is still under investigation. Additionally, it has certain cardiovascular and gastrointestinal side effects [22]. However, we remain confident in the potential of inhibiting MAO-B to prevent or treat AD, and we believe it also holds promise for the treatment of Parkinson’s disease and depression, while finding MAO-B inhibitors with minimal natural toxicity and side effects hold significant clinical value.

This study employed the TCMSP database to predict the BBB permeability of candidate compounds by evaluating their ability to enter the central nervous system (CNS) via the LogBB value (log[C]brain/[C]blood), where a LogBB > −1 is generally considered indicative of BBB permeability [23]. To further validate these predictions, the BOILED-Egg model available in the SwissADME database—based on molecular polar surface area (TPSA) and LogP—was also utilized, and the results suggested that the candidate compounds have the potential to cross the BBB [24]. It should be noted that BBB permeability is influenced not only by the physicochemical properties of compounds but also by complex factors such as active transport and metabolic stability. Incorporating quantum chemical analysis can further validate BBB permeability, as this approach provides a more in-depth examination of compound-target interactions and its inclusion may enhance the overall analysis [25]. Meanwhile, the molecular dynamics simulation results demonstrate that the complexes exhibit good conformational stability, indirectly supporting the potential for the candidate compounds to exert pharmacological effects after crossing the BBB [26].

After screening a series of natural compounds, we constructed a protein–protein interaction (PPI) network and analyzed small-scale subnetwork genomes using the molecular complex detection (MCODE) plugin, which included *MAO-B*, *AChE*, and *FAAH*. *FAAH* is also closely associated with Aβ deposition, suggesting that it plays a regulatory role in microglial function in Alzheimer’s disease-related pathological changes [27]. The decrease in ACh levels is closely related to cognitive decline in AD patients, while ferulic acid can inhibit the activity of acetylcholinesterase (AChE), thereby helping to increase ACh levels in the brain [28]. The interconnected roles of MAO-B, AChE, and FAAH highlight the multifaceted neuroprotective effects of ferulic acid and provide new insights into its therapeutic potential.

Factors contributing to poor drug performance in AD clinical treatment include target selection, late treatment initiation, and side effects. The structural polymorphism of Aβ makes the precise selection of drug targets more complex. At the same time, overemphasis on the Aβ hypothesis has hindered the emergence of diverse new targets [29]. The neuroprotective and anti-inflammatory functions of lingonberry can intervene in AD from multiple aspects [30]. In this study, molecular docking and dynamic simulation analyses revealed that ferulic acid acts as a potent inhibitor of MAO-B, suggesting a direct role in modulating neurotransmitter metabolism. Given existing evidence that polyphenols ameliorate metabolic dysfunction by enhancing insulin sensitivity, reducing blood pressure, and lowering blood glucose levels—thereby reducing the risk of AD—ferulic acid may mitigate AD pathology through multiple mechanisms [31]. These integrated findings not only expand our understanding of AD treatment mechanisms but also highlight the therapeutic potential of natural compounds, paving the way for future drug discovery and development.

## 4. Materials and Methods

### 4.1. Databases and Software

The following databases and software were used to build the network pharmacology and molecular docking: the Herb database (http://herb.ac.cn/), the PubChem database (https://pubchem.ncbi.nlm.nih.gov/), the SwissADME database (http://www.swissadme.ch/), the Traditional Chinese Medicine Systems Pharmacology Database and Analysis Platform (https://test.tcmsp-e.com/index.php), the SwissTargetPrediction database (http://swisstargetprediction.ch/), the DisGeNET database (https://disgenet.com/), the GeneCards database (https://www.genecards.org/), the OMIM database (https://www.ncbi.nlm.nih.gov/omim), the PharmGKB database (https://www.pharmgkb.org/), the CTD database (https://ctdbase.org/), the STRING database (https://cn.string-db.org/), Cytoscape version 3.8.1, the DAVID database (https://davidbioinformatics.nih.gov/home.jsp), the OECloud tools (https://cloud.oebiotech.com/#/), the Gene Expression Omnibus database (https://www.ncbi.nlm.nih.gov/geo/), the RCSB Protein Data Bank database (https://www.rcsb.org/), AutoDockTools version 1.5.7, PyMol version 2.6, and PoseView (https://proteins.plus/help/poseview). The access date for all databases is 26 February 2024.

### 4.2. Determination of the Main Phytochemicals of Lingonberry and Evaluation of Pharmacological Parameters

In this study, we retrieved 62 distinct compounds from the Herb database [32] and relevant literature [19], which are derived from the foliage and fruit of the lingonberry. The canonical SMILES strings for these compounds were obtained from the PubChem database. Thereafter, we performed RO5 screening using the SwissADME database [24], using the following parameters: molecular weight (MW) of less than 500 Daltons, a hydrogen bond donor (Hdon) count of 5 or fewer (including groups such as hydroxyl and amino), a hydrogen bond acceptor (Hacc) number capped at 10, a logarithmic lipid–water partition coefficient (LogP) ranging from −2 to 5, and a rotatable bond (Rbon) count not exceeding 10. These parameters are widely used to evaluate the oral bioavailability of compounds. The blood–brain barrier (BBB) permeability of the candidate compounds was predicted using the Traditional Chinese Medicine Systems Pharmacology Database and Analysis Platform (TCMSP). The evaluation was based on the LogBB value (log[C]brain/[C]blood), where a LogBB > −1 is generally considered indicative of BBB permeability [23]. By applying the aforementioned parameters, we effectively reduced the list to compounds that met the established criteria. The chosen compounds are scheduled for further investigation in the subsequent phases of this study.

### 4.3. Collection of Lingonberry–Related Targets

The SwissTargetPrediction database [33], a web server specialized in predicting targets for bioactive small molecules, was used to identify potential targets of the predominant phytochemicals in lingonberry. The canonical SMILES of these compounds were uploaded to the SwissTargetPrediction database, specifying Homo sapiens as the target organism. Targets identified with a prediction probability over 0.05 were selected and systematically catalogued using Microsoft Excel (version 2019) to manage data.

### 4.4. Construction of the Protein–Protein Interaction (PPI) Network

Genes associated with Alzheimer’s disease were searched across five common disease databases using specific criteria. In DisGeNET [34], genes with a Score_gda greater than 0.1 were identified. In GeneCards [35], genes with a relevance score over 10 were selected. OMIM [36] selected genes with a confirmed Entrez Gene ID. In the PharmGKB [37] database, relevant genes were included in the AD-related genes set using specific keywords. In the CTD database, genes with the direct evidence tags “marker/mechanism” were selected. The genes collected from these databases were then curated to remove duplicates. A Venn diagram was created using jvenn [38] to visualize the overlap of these gene sets. The protein–protein interaction (PPI) network was constructed using the STRING platform [39], with the species set to “Homo sapiens”. Subsequently, the molecular complex detection (MCODE) tool in Cytoscape version 3.8.1 software was used to identify key modules within the PPI network, using default parameters.

### 4.5. Gene Ontology (GO) Functional and Kyoto Encyclopedia of Genes and Genomes (KEGG) Pathway Enrichment Analysis

We performed Gene Ontology (GO) and KEGG pathway analyses on the target genes using the DAVID [40] platform. Subsequently, we utilized the OECloud tools [41] to visualize the analysis results. Through careful examination and interpretation of the data, we gained a deeper understanding of the functional enrichment and pathway affiliations of the genes, providing valuable insights for exploring potential therapeutic targets for AD.

### 4.6. AD Chip Data Collection

We queried the Gene Expression Omnibus (GEO) database using the keyword “Alzheimer’s disease” to identify relevant gene expression datasets. After rigorous screening, we selected the GSE117585 dataset for analysis. This dataset is based on the Affymetrix Human Genome U133 Plus 2.0 Array (HG-U133_Plus_2) platform and includes five samples from patients with sporadic Alzheimer’s disease (SAD) and six normal control (NL) samples. To ensure data consistency and comparability, we performed quantile normalization using the normalizeBetweenArrays() function in the limma package (version 3.44.0) [42] in R (version 4.1.2). This method standardizes gene expression distributions across all samples, reducing systematic biases and enhancing data reliability. Differential gene expression analysis was conducted using limma, with stringent selection criteria of log2|FoldChange| > 1.5 and *p*-value < 0.05 to identify differentially expressed genes (DEGs). The intersecting DEGs were further processed for downstream analyses. To ensure data quality, we carefully curated the gene expression data by removing duplicate entries and verifying dataset integrity.

### 4.7. Molecular Docking Analysis

We utilized the molecular docking technique to elucidate the interactions between the key target and bioactive compounds. The structures of the targets, based on X-ray crystallography, were sourced from the RCSB Protein Data Bank (PDB) [43], a public database that archives 3D structural data of proteins and nucleic acids. We specifically chose the crystal structure of MAO-B, as determined by Binda et al. [44], which has a resolution of 2.12 Å. For ligand selection, we selected ferulic acid, benzoic acid, erythrodiol, and p-coumaric acid, all of which are elements found within the intersection of our gene set. The three-dimensional structures of these compounds were obtained from PubChem. To prepare the protein structure for docking, we used PyMOL to remove all water molecules and co-crystallized ligands, thereby eliminating potential non-specific interactions that could compromise docking accuracy. Further optimization was performed using AutoDockTools, where hydrogen atoms were added to refine the hydrogen bonding network, Gasteiger charges were assigned to ensure accurate electrostatic interaction calculations, and AutoDock 4 atom types were defined to adapt the receptor to the docking algorithm. For the binding site selection, we employed a grid box covering the entire protein, allowing ligands to freely explore all potential binding sites. To validate our docking protocol, we employed selegiline, a known MAO-B inhibitor, as the positive control [45]. The docking results showed that selegiline exhibited a binding energy of −8.12 kcal/mol, forming four hydrogen bonds with an RMSD of 0.98 Å, confirming its strong binding affinity to the active site of MAO-B. Additionally, to ensure a robust comparative analysis, we selected compounds with weaker docking scores (binding energy > −5 kcal/mol) as negative controls, thereby assessing the relative binding capacity of our candidate molecules.

All docking simulations were conducted using AutoDock (version 1.5.7) [46], with default parameters applied. The docking results were analyzed through PyMOL (version 2.6) [47], focusing on binding energies, hydrogen bond interactions, and root mean square deviation (RMSD) values. Furthermore, to enhance the visualization and interpretation of ligand–receptor interactions, PoseView was employed to generate two-dimensional interaction diagrams, highlighting the key catalytic residues involved in ligand binding.

### 4.8. Molecular Dynamics Simulation

**Figure 7:**
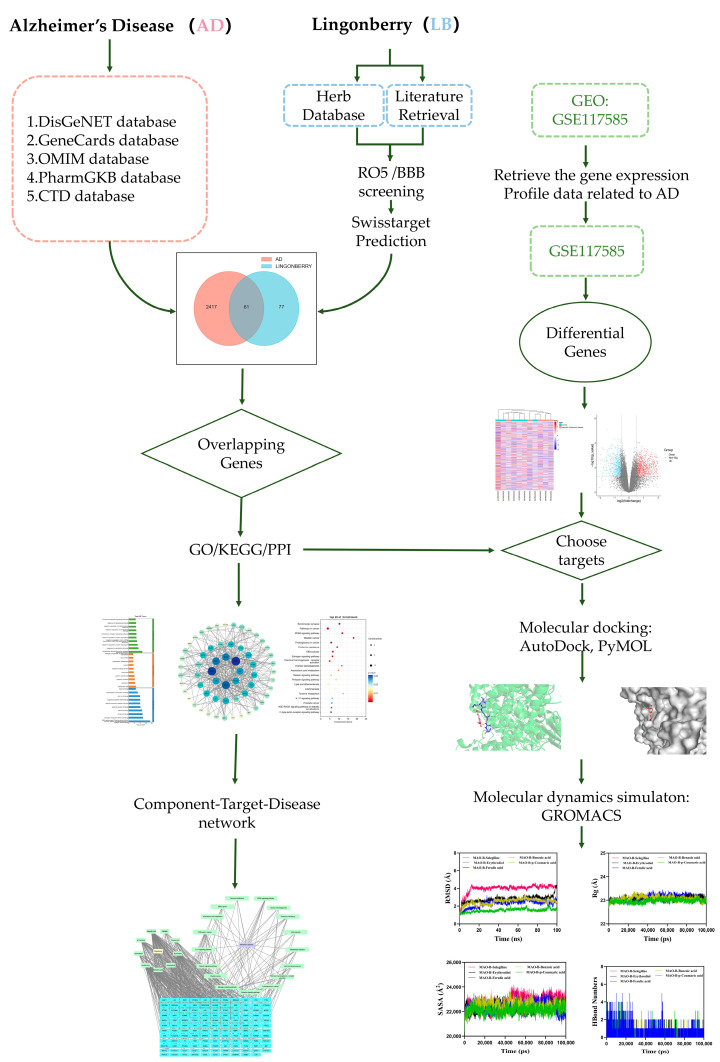
The flow chart of this research.

Molecular dynamics (MD) simulations of the protein–ligand complex were performed using GROMACS 2023 for a total duration of 100 ns. The protein was parameterized with the CHARMM36 force field [48], while the ligand topology was generated using the GAFF2 force field. The complex was placed in a cubic simulation box under periodic boundary conditions and solvated with TIP3P water molecules [49]. Electrostatic interactions were treated using the particle mesh Ewald (PME) method, while the Verlet algorithm was employed for non-bonded interactions. The system underwent equilibration in the NVT and NPT ensembles for 100,000 steps, with a coupling constant of 0.1 ps, for a total of 100 ps. A cutoff of 1.0 nm was applied for both van der Waals and Coulomb interactions. Finally, a 100 ns MD simulation was conducted at a constant temperature of 300 K and a pressure of 1 bar using GROMACS 2023. The flow chart of this study is shown in Figure 7.

## 5. Conclusions

In summary, our study uses network pharmacology and molecular docking analyses to discuss how lingonberry improves Alzheimer’s disease through multiple pathways and targets. It also demonstrates at the molecular level the potential of its active component, ferulic acid, to act as an inhibitor of MAO-B and thereby contribute to the improvement of Alzheimer’s disease. This is highly significant for the potential therapeutic application in AD. These results underscore the therapeutic potential of lingonberry, as its abundant active compounds modulate various AD risk factors and offer new insights into controlling disease progression.

## 6. Patents

The Role of Wild Cranberries in Improving Memory Function in Alzheimer’s Disease Patients (202410140430.7).

## Figures and Tables

**Table 1:** Binding energy, hydrogen bond formation, and RMSD for molecular docking of five compounds with MAOB.

	Ferulic Acid	Benzoic Acid	Erythrodiol	p-Coumaric Acid	Selegiline
Binding energies	−5.26	−4.96	−7.78	−5.01	−8.12
Hydrogen bond formation	3	0	0	3	4
RMSD	1.146	1.078	0.001	1.224	0.98

## Data Availability

Data is contained within the article.

## Abbreviations

The following abbreviations are used in this manuscript:

AD	Alzheimer’s disease
MCI	mild cognitive impairment
Aβ	amyloid β
PPI	protein–protein interaction
MAO-B	monoamine oxidase B
MW	molecular weight
Hdon	hydrogen bond donor
Hacc	hydrogen bond acceptor
LogP	logarithmic lipid–water partition coefficient
Rbon	rotatable bond
BBB	blood–brain barrier
TCMSP	Traditional Chinese Medicine Systems Pharmacology Database and Analysis Platform
MCODE	molecular complex detection
GEO	Gene Expression Omnibus
PDB	RCSB Protein Data Bank
RMSD	root mean square deviation
Rg	radius of gyration
SASA	solvent-accessible surface area
RMSF	root mean square fluctuation
CNS	central nervous system
PPs	polyphenols
CTD	component–target–disease
